# Identifying maternal needs following childbirth: A qualitative study among mothers, fathers and professionals

**DOI:** 10.1186/s12884-017-1398-1

**Published:** 2017-07-03

**Authors:** J. Slomian, P. Emonts, L. Vigneron, A. Acconcia, F. Glowacz, J. Y. Reginster, M. Oumourgh, O. Bruyère

**Affiliations:** 1 Department of Public Health, Epidemiology and Health Economics and Support Unit in Epidemiology and Biostatistics, University of Liège, Avenue Hippocrate 13, Bât. B23, 4000 Liège, Belgium; 2 Department of Obstetrics and Gynaecology, CHU Liège, Avenue Hippocrate 13, Bât. B23, 4000 Liège, Belgium; 3 Wallonia e-health Living Lab, The labs, Parc scientifique du Sart-Tilman, Rue Bois Saint Jean 15/1, 4102 Liège, Belgium; 4 Department of Psychology, University of Liège, Quartier Hôpital, Avenue Hippocrate 13, Bât. B23, 4000 Liège, Belgium; 5 Department of Motricity Science, University of Liège, Liège, Belgium; 6 Public Health Department, Bone and cartilage metabolism Department, CHU Liège, Quai Godefroid Kurth 45, 4000 Liège, Belgium

**Keywords:** Postpartum period, Needs, Information, Psychological support, Sharing experience, Practical support

## Abstract

**Background:**

Pregnancy and childbirth are two critical stages in a woman’s life. Various studies have suggested that psychological distress is common during the year after childbirth. The objectives of this exploratory study were (1) to explore the needs of mothers in the year following childbirth; (2) to compare these needs between mothers who did not have the feeling of living a psychological disorder or a depression and mothers who lived a psychological disorder or had the impression of living a depression; and (3) to compare the needs expressed by mothers with the perception of professionals and fathers about the mothers’ needs.

**Methods:**

First, we proceeded to 22 individual qualitative interviews followed by one focus group, with mothers, with and without experience of psychological distress. Then, we conducted 2 focus groups: one with professionals and one with fathers.

**Results:**

Needs of mothers after childbirth have been indexed in four categories: need of information, need of psychological support, need to share experience, and need of practical and material support. Women do not feel sufficiently informed about this difficult period of life. They do not feel sufficiently supported, not only from a psychological point of view but also from a more practical point of view, for example with household chores. They need to share their experience of life, they need to be reassured and they need to feel understood. It seems that some differences exist between mothers’ and professionals’ experiences but also between mothers’ and fathers’ experiences.

**Conclusion:**

Young mothers apparently feel a lack of support at different levels in the year following childbirth. This study provides ways to meet women’s needs and to try to prevent the risk of postpartum psychological distress during this period of time.

## Background

Pregnancy and childbirth are two critical stages in a woman’s life. The postnatal period is a stressful time in the life of a woman with sudden and intense changes in women roles and responsibilities [1–3]. The majority of the women seem to have anxieties and fears around early parenting and their changing roles [4]. Women are often concerned about the safety of their new baby, and lack self-confidence as new mothers and in their own ability to care for their baby. Women therefore need to be surrounded by those who will emotionally support them in this transition to parenthood [5, 6].

Psychological distress therefore appears to be common after childbirth. Indeed, women can experience a range of psychological problems after birth, including anxiety, post-traumatic syndrome, adjustment disorders or depression [1, 7, 8]. Prevalence rate of maternity blues among women varies between 5 and 80% depending on the diagnostic criteria [2]. Over a third of mothers are at risk of developing early postpartum affective disorders [9]. Characterized disorders such as phobic disorders affect 10 to 16% of women after childbirth [2]. The prevalence of postpartum depression, evaluated by self-reported questionnaires, in developed countries varies from 1.9 to 82.1%, with the lowest reported in Germany and the highest in the United States [3, 10]. In developing countries, the prevalence varies from 5.2 to 74.0%, with the lowest prevalence reported in Pakistan and the highest in Turkey [3].

Throughout this paper, we will focus also on mothers’ depression and/or major psychological distress. The aetiology of depression and psychological distress after childbirth remains unclear [11] but several risk factors have been identified. Norhayati et al. categorized these risk factors into physical and biological (e.g. poor physical health), psychological (e.g. antenatal depression, stressful life events), obstetric and paediatric (e.g. unplanned pregnancy, emergency caesarean section, stress induced by the baby), socio-demographic (e.g. young maternal age), and cultural groups (e.g. low social support) [3, 8]. Therefore, we hypothesize that mothers’ depression and/or major psychological distress could be due to unmet needs of mothers during the perinatal period.

In addition, in various countries, health authorities have decided, mainly for economic reasons, to implement a new health reform consisting into reducing the length of stay in maternity units after childbirth [12]. This measure might have changed or increased women’s needs after childbirth. To our knowledge, very few studies have assessed mothers’ needs in the postnatal period [13–19]. Our research question is therefore: “To what extent having experienced an episode of psychological distress in the year following childbirth could influence the needs of mothers during this period?”. The objectives of this exploratory study were (1) to explore the needs of mothers in the year following childbirth; (2) to compare these needs between mothers who did not have the feeling of living a psychological disorder or a depression and mothers who lived a psychological disorder or had the impression of living a depression; and (3) to compare the needs expressed by mothers with the perception of professionals and fathers about the mothers’ needs. Our long-term goal is to examine if it is possible to reduce the psychological distress in the postnatal period by better meeting the needs identified in this study.

## Methods

**Fig. 1:**
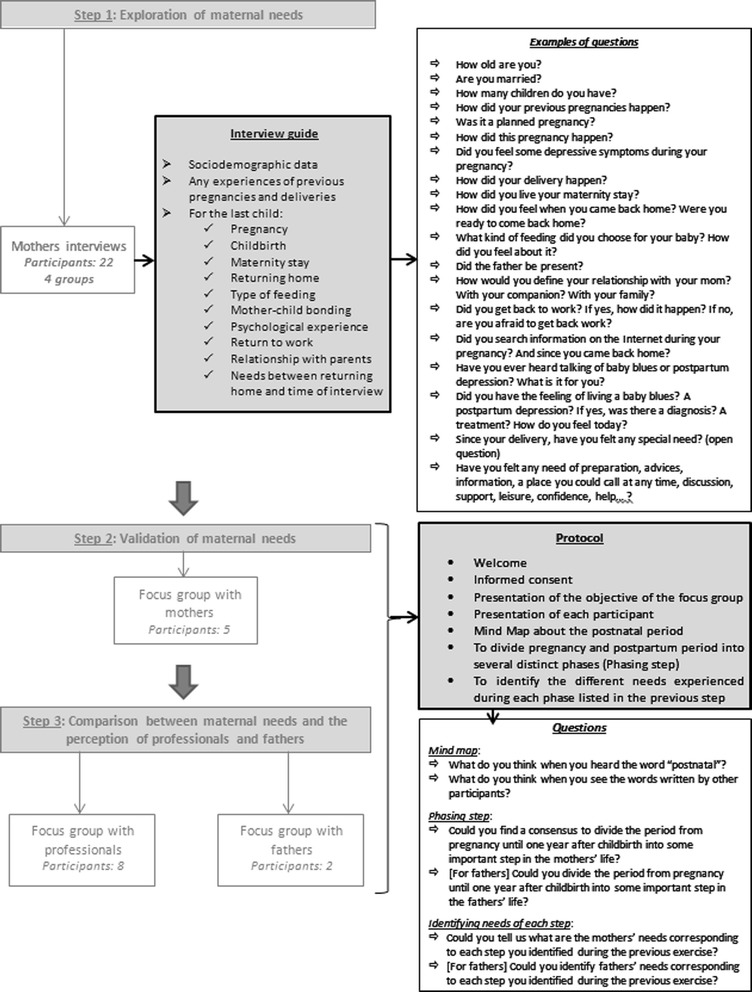
Qualitative steps to identify needs of mothers in the year following childbirth

A multi-stage qualitative study was undertaken, which involved individual and focus group interviews of mothers, and focus interview of health professionals and fathers. Each of the stage undertaken in this study is represented in Fig. 1.

### Individual interviews

The first step (step 1) of this study consisted in conducting individual interviews with four different groups of mothers to explore the maternal needs:

- a group of mothers, who had given birth 4 to 6 weeks earlier, not having the feeling of living a psychological disorder or depression (group 1);
- a group of mothers, who had given birth 4 to 6 weeks earlier, showing a psychological disorder or having the impression of living a depression (group 2);
- a group of mothers, who had given birth 10 to 14 months earlier, not having the feeling of having experienced a major psychological disorder after childbirth and for whom postnatal depression had not been diagnosed (group 3);
- a group of mothers, who had given birth 10 to 14 months earlier, having experienced a major psychological disorder after childbirth (undiagnosed) or for whom postnatal depression had been diagnosed (group 4).

All women who met the criteria of one of the four above groups and who agreed to participate were eligible for this study. Events of psychological disorder or depression were assessed through personal experience of the participants but also with the EPDS Questionnaire [20]. Exclusion criteria were: twin pregnancy, foetal death in utero, very premature childbirth (<34 weeks of gestation), foetal-pathologies. We expected to interview 10 mothers by group of study (*n* = 40).

Recruitment of participants was done through various ways. On one side, we visited the two biggest maternity hospitals of the city of Liège (Belgium) to talk about the study to new mothers: those who were interested in the study were contacted 3 to 4 weeks later to schedule an interview (groups 1 and 2). On the other side, the study was also posted on social networks: Facebook, the websites of the WeLL (Wallonia e-health Living Lab) and of the AlterNative (platform for a respected birth). Posters were also placed in different paediatric and gynaecological services. Participants, who expressed their interests, were contacted by telephone. More information on the study was given and if they agreed to participate in the study, an appointment for the interview was fixed (groups 3 and 4).

The face to face interviews took place at the mothers’ home. Each interview began with a reminder of the context of the research. The rights of the participant and the ethical and legal context of the research were also reminded. A written description of the research was provided to the participant and informed consent was collected. The interview then started. The researcher had an interview guide which is presented in Fig. 1. The interview guide has been prepared and constructed by the main researcher who is a midwife and a Ph.D. Candidate in Public Health with the contribution of a psychologist, a gynaecologist and an epidemiologist.

### Focus groups

The second step (step 2) of this study consisted in the validation of maternal needs. A focus group was therefore organized with mothers willing to share their opinions and experience. We wanted to bring women together in order to discuss their respective needs, to identify links between their experiences and hopefully to bring forward some new ideas. Recruitment of participants was mainly done through social networks (Facebook, the website of WeLL, AlterNative) and by word of mouth. Participants of interviews were also contacted to participate in these focus groups.

The third step (step 3) of this study consisted in a comparison between maternal needs and the perception of professionals and fathers. Two other focus groups were therefore organised and conducted. One focus group was done with some professionals, orbiting mothers of children aged 0 to 1 year, to get an external opinion and to confront perception of professionals with perception of mothers. Another focus group, this time with some fathers, was conducted after the professionals’ focus group. Indeed, opinions of mothers and professionals about the place of the father were different: so, we sought their opinion. Concerning the focus group with professionals, participants were contacted on account of their specialty (gynaecologist, midwife, paediatrician, general practitioner, psychologist, Medical-Social Worker of the ONE (“*Office de la Naissance et de l’Enfance*”: Belgian Office of Birth and Childhood), and nursery nurse. For the focus group with fathers, recruitment of participants was mainly done through social networks (Facebook, the websites of WeLL and AlterNative) and by word of mouth.

The three focus groups were conducted using the same protocol which is presented in Fig. 1. The focus groups’ protocols were conceived by the main researcher with the contribution of the WeLL which is a living lab specialised in the use of co-creative methods in qualitative researches. All focus groups were held in the same premises: those of the WeLL. First, participants were asked to introduce themselves to each other and the search topic was presented. Then, we began the exploration with a mind map. The mind map consisted of mapping participants’ thinking on the theme: they had to think about the term “postnatal”. A first participant would then note a first word to which he thought when he heard the word “postnatal”; a second participant would then note a second word and so on. Participants could also bounce off their own words or words noted by others. All the advantages of including the mind mapping in qualitative research are described in the article of *Burgess-Allen and Owen-Smith* [21]. Once completed, the next step of the focus group was to divide pregnancy and postpartum period into several distinct phases which were experienced by the majority of mothers. The last step was to identify the different needs experienced during each phase listed in the previous step.

### Analyses

Interviews and focus groups were audio-recorded, using a Dictaphone, then transcribed verbatim and thematically analysed. Data analysis began before all interviews were conducted: the researcher could therefore control for topic saturation [22]. Topic saturation occurred after the 17th interview. Interviews were therefore completed with only 22 of the 40 participants expected. An identification number was allocated to each interview participant. To ensure confidentiality, all information allowing any identification was removed from the transcripts. Transcripts were checked twice (2 researchers: the main research and a second researcher who was midwife and an epidemiologist too) to ensure the accuracy of the transcription before encoding. During focus groups, handwritten notes were also captured and were analysed afterwards to help identifying the different themes.

Qualitative content analysis was conducted according to Neale [23]. Transcripts were systematically coded by topic. The categories of needs were developed inductively from the material of the first interviews. Indeed, the transcripts were summarized and then, some key words were labelled and classified into groups of similar issues (=categories). Categories and sub-categories were then attributed to transcripts passages. These passages were also gathered under common themes. In the results section, some direct quotes extracted from the interviews or focus groups, are provided to illustrate each theme. When necessary, they are contextualized by the parity (number of children born) and/or the presence of depression of the informant, or by the focus group involved.

The analysis and the coding were conducted on one hand, by the first researcher without using any computer software; and, on the other hand, by the second researcher using NVivo 11 for Windows (qualitative data analysis computer software package produced by QSR International). The coding was finally discussed between the two researchers to ensure the validity and the credibility of the results.

## Results

### Participants

Twenty-two women, between 26 and 42 years old, participated in the interviews: 10 women in group 1, 5 women in group 2, 2 women in group 3 and 5 women in group 4. Ten of them were mothers for the first time, ten of them for the second time and two of them for the third time. Interviews lasted between 30 min and 2 h depending on the participants. The sociodemographic and pregnancy profiles of the interview’s respondents are presented in Table 1.

**Table 1:** Sociodemographic and pregnancy profiles of the interview’s participants

Variable	Group 1 (*n* = 10)	Group 2 (*n* = 5)	Group 3 (*n* = 2)	Group 4 (*n* = 5)	Total population (*n* = 22)
Age (years; mean ± SD)	33.3 ± 5.27	30.6 ± 4.56	27.5 ± 0.71	29.4 ± 3.21	31.3 ± 4.70
Marital status (n (%))					
Married/In couple	9 (90.0)	3 (60.0)	2 (100)	5 (100)	19 (86.4)
Single/Divorced/Separated	0 (0.00)	1 (20.0)	0 (0.00)	0 (0.00)	1 (4.55)
Other	1 (10.0)	1 (20.0)	0 (0.00)	0 (0.00)	2 (9.09)
First pregnancy (n (%))					
Yes	5 (50.0)	1 (20.0)	1 (50.0)	3 (60.0)	10 (45.5)
No	5 (50.0)	4 (80.0)	1 (50.0)	2 (40.0)	12 (54.5)
Gender of children					
Female	6 (60.0)	4 (80.0)	1 (50.0)	3 (60.0)	14 (63.6)
Male	4 (40.0)	1 (20.0)	1 (50.0)	2 (40.0)	8 (36.4)
EPDS score after 1 month (/20; mean ± SD)	7.40 ± 2.32	13.6 ± 3.51	11.5 ± 6.36	19.5 ± 9.85	11.6 ± 6.61
EPDS score after 6 months (/20; mean ± SD)	N.A.	N.A.	10.0 ± 0.00	10.0 ± 6.48	10.0 ± 5.02
EPDS score after 1 year (/20; mean ± SD)	N.A.	N.A.	8.00 ± 1.41	6.50 ± 4.65	7.00 ± 3.74

*N.A.* not applicable

Concerning the focus groups, 4 “first-time” mothers and 1 “second-time” mother participated in the mothers’ focus group. Two out of the five mothers lived a postnatal depression. There were 8 participants in the focus group with professionals: 1 gynaecologist, 1 midwife, 1 general practitioner, 2 psychologists, 2 Medical-Social Workers of the ONE and 1 nursery nurse. Finally, two fathers – both fathers for the second time –participated in the fathers’ focus group.

### Needs

A report was made after each step analysis. A synthesis of all these analyses was then built to highlight major categories of needs of mothers in the year following childbirth. It is this synthesis that is presented in this article. The needs of mothers who experienced a depression and the needs of those who did not experience a depression were also compared. No difference was found between these two populations. All women seemed to have the similar needs but at different level of intensity. We listed needs of mothers after childbirth into four categories: need of information, need of psychological support, need to share experience and need of practical and material support. Each of these categories has then been subdivided into more specific needs (Table 2).

**Table 2:** Identified needs of mothers during the year following childbirth and their sub-categories

Identified needs & needs’ sub-categories	Description / Examples
Need of information	
Existing services	-Post-natal physiotherapy -Independent midwives -Osteopaths -Reimbursement system available -...
Medical information	-“What should I do?” (In case of fever, jaundice, colic, constipation, for growth spikes, breastfeeding, first teeth ...): “when should I worry and consult?” -Need for regular medical visits: especially at the beginning of the return at home
Practical advices	-Furnishing of the room -Breastfeeding -Teat or not? -Night management -Household management -Couple and sexuality management -Nursery: how it happens?
Social idealization of the motherhood vs reality	-Be forewarned of the difficulty of adaptation -Variable adaptation time -Feeling outdated could be normal -Variable time of taming of her child
Administrative information	-Declaration of birth -Family allowances -Rights -Reimbursement system for some services -Legislation (e.g. for to work)
RELIABILITY of the information	-Reliable, coherent and real information -Recommended by professionals
Need to share experience	
Need to compare their own experience with other experiences and to be reassured	Different source of sharing: -Father -Family -Friends -Other Mothers (friends, social networking, discussion group, organised group session): some mothers prefer to talk with Experienced mothers and other mothers prefer to talk with woman in the same situation (baby of the same age) -Health professionals
Fight against the loneliness feeling	-Feeling of isolation and abandonment of a lot of mothers (especially for the first baby)
Activities / leisure	-Unpack / blow -See other people -Activities between young mothers to discuss the experience
Need of psychological support	
Psycho-relational	-Need to be surrounded (experiencing social isolation ± strong) -Need to be reassured -Need to hear that they are doing well, that they are good mothers (skills/self-esteem) -Father’s place -Need to be understood: empathy of the entourage and the professionals
Pathological	➢Mother’s psychological distress and/or Pathology revealed in children -Need of psychologists, psychiatrist or other professionals if necessary; medicines (e.g. antidepressants) ...
Need of practical and material support	
Services	-Domestic help: cleaning, ironing, cooking, shopping ... -Involvement of the father is required -Help to have time to take care of her baby (not the opposite: help to take care of the baby while the mother cleans!)
Economical	-Financial aid -System of “service vouchers” reimbursed in the first weeks after childbirth

### Need of information

Interviews and focus groups seemed to show a great need for information among women in the year following childbirth.

Indeed, most of the mothers denounced social idealization around motherhood. They insisted on the need to be warned of the difficulty of becoming a mother. This is especially true for first-time mothers. They said that it has not always been easy to adapt to their new role and that they often felt overwhelmed by events. There is such an idealization of motherhood that they did not dare to speak or complain about their problems. It is only later that they discovered that many other mothers have found themselves in the same situation and that difficulties of motherhood are still taboo. They wished that mothers and society talked more openly and in a less perfect way of motherhood.

*“Although we know that it may be a bit difficult, they (the mothers) are there with their photos, with their baby. On the photos, everything is peachy ... we finally believe that it’s all true! I thought it would be just like on TV, I would go walking with my baby, we would almost have discussions; almost like a little friend! But in fact, not at all! The first 3 months, there is really no exchange” (Interview 13).*

*“Beware of misconceptions: we’re not in a heavenly thing, it is hard!” (Focus group with mothers).*

*“Even so during pregnancy, we are in something else and we’re probably not ready to hear it, we would like to be prepared, in late pregnancy, about what will happen after (first baby)” (Focus group with mothers).*

Most first-time mothers said they would have liked to be better prepared for the postpartum period. They would have needed other persons, professionals or family, to anticipate their needs and questions because they did not know what to expect. Moreover, they tended to listen to whatever they were told about how to be the best mother, but then they realized that there are as many different opinions as there are professionals.

*“For a second baby, we know how to do but for a first baby, no one ever tells you! In fact, the phrase I heard the most during my pregnancy was “you'll see when the baby will be there”. What do you want me to see when the baby is here? You have to anticipate a little!” (Interview 2).*

*“We do not give a lot of credit to mums. For the first baby, I wasn’t listening to myself because I was listening to the “white blouse” (Author’s note: corresponding to health professionals). I took their advice as if it was an exact science but I should have listened to me. I was so stupid! I'm the mother. This is my child. I know better anyway ...” (Interview 11).*

The majority of mothers, whether first-time mothers or not, expressed their need for “medical information”. When all goes well, they need that health professionals confirm that all is well. Regular doctor visits help to reassure mothers about their own health and that of their child or children. Likewise, when there is a specific problem (feeding problems, fever, jaundice, growth peaks, colic, constipation, teething ...), they would be better informed whether to consult a professional. Even mothers who already have one or more children need information on what they have not yet experienced with their first child. All the more reason, first-time mothers need even more medical information.

*“I would have liked to have information about growth spurts, colic ... explanations about baby care: what can I give or not, additional water or not, pacifier or not ... There were times when he drank nothing: what should I have done?” (Interview 6, 1* ^*st*^ *baby).*

*“I would have liked if someone had talked to me about infant acne and about the weird sounds that a baby makes because sometimes it's scary” (Interview 15, 3* ^*rd*^ *baby).*

*“I think we do not have enough information on the babies’ food evolution (Authors’ note: meaning “introduction of novel foods”): if you don’t ask questions, you don't have information” (Interview 16, 2* ^*nd*^ *baby).*

When leaving the maternity ward, many mothers (and fathers) would have liked to have more practical advice on the management of homecoming and on how and what to do with their child (first outing with the baby, practical advice on breastfeeding, pacifier, nights management, household management, management of the couple and sexuality, information on childcare facilities ...).

*“We would have liked to be told what activities we could do with the baby, when we could go out for the first time with him” (Focus group with dads).*

*“I think we are told a lot of things when on maternity ward but we don’t remember them. We need it to be more practical; perhaps medical or technical “How-to” sheets” (Interview 16).*

A lot of women felt a lack of information about available services during the postpartum period (independent midwives, home health service, postnatal physiotherapy, osteopathy, possible reimbursement system ...). They also denounced the difficulty to find this kind of information and would have liked it to be centralized in one convenient place.

*“We didn’t know any osteopath who takes care of babies; nobody around us, we were the first to have a baby” (Interview 13).*

*“There is a lack of information on what exists: we have to look, ourselves, for a physio although it is prescribed by the gynaecologist! By chance, I’ve discovered physio group sessions, it was very good but you have to know that it exists!” (Focus group with mothers).*

Some mothers also complained about the lack of administrative information on declaration of birth, family allowances, rights of mothers and parents, reimbursement system for certain services, professional legislation, etc.

*“I can’t return to work either morally or physically: I work far away and I don’t have a childcare solution. So I looked into the decrees to find a solution, I’m still waiting ... I would have liked to receive advice!” (Interview 5, high risk of PPD).*

*“[…] We need some administrative information: when and where do we register the birth, information on the possibility to do things in advance (during pregnancy) ... I didn’t even know that childbirth allowance existed” (Focus group with mothers).*

The need for information is particularly ruled by the need for reliable sources of information. It seems that hospitals leaflets sometimes give contradictory information. Websites and forums exist but mothers denounced the lack of reliability of these sources. They need it to be consistent and reliable and would like to find information that would be controlled by professionals.

*“The Internet is really nice. It's pretty easy and it allows you to do some research when you have questions. But you can’t be sure to find reliable information” (Interview 7).*

*“We have lots of reading, I don’t really have the energy to read everything so I select some of them. I realized that from one booklet to another, different things are told. The ONE and the hospital where I gave birth do not say exactly the same thing either...” (Interview 11).*

*“Someone passed me some books: I read, month by month, what’s going to happen, it is interesting” (Interview 19).*

### Need of psychological support

Even when there was no big problem during the postpartum period, some women felt isolated after giving birth. They expressed their need to be supported because of this social isolation.

*“Mums should have a reference person, even already during pregnancy. Plus, mums would at least have a few visits and they would actually be less isolated” (Interview 11).*

*“We have a couple of friends who do not want children. I think it bothers them. We felt a gap already during pregnancy. I think it bothers them because we are no longer available” (Interview 18).*

Most mothers need to be reassured and to hear that they do well and that they are good mothers. Mothers who already have a child may feel less this need but some of them still need to be encouraged in their role of mothers.

*“I was really happy when she (ONE’s medical-social worker) came home; happy to have someone who comes and looks if everything goes well, just gives me confidence” (Interview 8, 1* ^*st*^ *baby, she’s a midwife).*

*“For monitoring breastfeeding, a midwife came home and weighed my baby. She said “so, you see that it works well!” Then I told myself that I was not doing so bad. At the beginning, I would have liked that someone had come more regularly because to wait one week is sometimes too long” (Interview 6, 1* ^*st*^ *baby).*

Many mothers also need to be understood in their situation by their family and by professionals.

*“Mom, Dad, understand that I don’t want you to come now: not because I don’t want to see you but because I'm exhausted!” (Interview 1).*

*“Depression, we are never far away!” (Focus group with mothers).*

*“Leaflets on maternity wards speak about babies not about mums ... Mothers are forgotten!” (Focus group with mothers).*

Fathers play a central role in the psychological well-being of the mothers. Some couples had arguments because of the new organization. Many mothers felt supported by their partner and said it was great to chat with them. Other mothers would have liked them to be more considerate and present.

*“I had a lot of help from the dad; it helped me a lot to be able to rely on him” (Interview 10).*

*“My husband supported me but he should have told me the truth: he saw that it was hurting me, I really wanted that damn breastfeeding, I wanted it at all costs ...” (Interview 2).*

Mothers who did not have a close or available family often felt this as a lack. The same applied when the father was not present (single mothers).

*“I tell myself that I didn’t have any luck with their dad. I would have liked to have someone who was there with me ... It’s hard” (Interview 21, absent father).*

*“If I had a normal family, I would have wanted advice, but I have none! I can’t count on them” (Interview 5).*

In situations where a medical problem arose, for example a state of psychological ill-being of the mother or the child being actually ill, the need for a psychological support became a medical need. In these cases, mothers needed the help of a professional, a psychologist or a psychiatrist, or even of some medication (e.g. antidepressants).

*“I told everyone that it was a bad delivery. Nobody was listening when I was explaining that it was wrong, that I wasn’t well. I would have liked that someone told me that I was losing control, I was falling ... (into depression)” (Interview 2).*

### Need to share experience

Most of mothers needed to share their experience, of being a new mother, with family or friends. Many mothers preferred to discuss with women who are already mothers (in family or friends network). Indeed, they said they need to compare experiences with other mothers to see if they were experiencing the same thing. They needed to be reassured about their mother’s skills. This sharing of experience allows them to see whether what they are experiencing is normal or not.

*“Is it normal to have the baby blues? Is it normal that I am still tired? Is it normal that one day, I'm very well and the next day, I’m in pain? Should I take a painkiller?” (Interview 11).*

*“Breastfeeding is sometimes very guilt-inducing because I do not always know if she (the baby) has eaten enough, for example. It's nice to have people around: friends who are already mothers, midwives, ONE medical social workers, paediatricians ... all contact-persons who helps you to stop feeling guilty” (Interview 7).*

Some women expressed their preference to talk to women who have children of similar age to compare experiences and evolution. Other women preferred chatting with mothers who have older children because they considered that these women have more experience and can better advise.

*“I was lucky because there are some mums around me: either mums who have had several children and who reassured me (“it is normal, don’t worry about that”); or mothers who gave birth at the same time as me and who are going through the same nightmares” (Interview 5).*

Being supported and sharing these experiences help to fight against the feeling of loneliness and isolation.

*“I am the first to have a baby among my friends but I think they will only realize that they were little present when they’ll have children of their own” (Interview 8).*

*“Different friends come to visit almost every day probably exactly because they’d experienced this isolation too” (Interview 11).*

Some women who were the first to have a child among people around them or who were isolated were more inclined to turn to social networks or discussion groups. Nevertheless, many women found these groups unreliable.

*“I think that social networks can be good because sometimes there are things that women are embarrassed to say and that they dare to write on the Internet (because of nicknames). They can share their true feelings or what really happened” (Interview 15).*

*“I'm on a webgroup of mums. We have children of all ages. Simply saying “how is it going with you?” It helps a lot” (Interview 5).*

Many women also expressed their need of a hobby to unwind, see other people and talk about other things. Some women have suggested the idea of organizing leisure activities among young mothers in order to spend time of their own but also to be able to discuss their experiences with other mothers.

*“Just to go out for an hour, that would be good!” (Interview 9).*

*“Just to take a breather, I miss that. I’m thinking about it but I still don’t know how to do it. It would be good to have someone who could look after your baby to enable you to have some leisure” (Interview 6).*

*“It’s difficult as long as I’m breastfeeding” (Interview 10).*

### Need of practical and material support

All mothers seemed to need help in managing the household: for cleaning, ironing, cooking, shopping, nights with the baby ... The involvement of the spouse seems necessary.

*“We should have a family support worker during the first months, to get the time to get organized” (Interview 20).*

*“I would like him (father) to help a bit when he gets home from work or when he’s on holiday” (Interview 1).*

Many women said seeking this kind of help to be able to have some time to enjoy being with the baby and to take care of themselves.

*“[...] My mother or my stepmother told me they were coming, but they were coming to take care of the baby while I was cleaning; I would have liked it to be the other way round” (Interview 1).*

*“I would like someone to look after the baby so I could sleep one hour” (Interview 5).*

This help would also encompass an economic pole that is to say financial aid. The idea of a “service voucher” system that would be reimbursed for the first weeks after birth was discussed several times. However, some women did not like the idea of having someone who would come to do their housework.

*“In the beginning, an adapted service would be good; the time to settle” (Interview 1).*

*“A service voucher system reimbursed like for twins” (Interview 6).*

### Comparison between maternal needs and the perception of professionals and fathers

There appears to be a discrepancy of opinion between professionals and mothers. Professionals are more concerned about the needs during pregnancy than those after the delivery. Professionals think that *“there is enough information for women after childbirth; mothers just need time to assimilate it”,* while women acknowledge to be “*suitably cared for during pregnancy and well prepared for childbirth”* but deplore that *“there is nothing”* after the delivery *(Author’s note: Mothers deplored feeling alone after the delivery. Indeed, there was much less medical monitoring for the mother after delivery while, during the pregnancy, they had regular appointments with their gynaecologist. Mothers have the impression that medical appointments after childbirth are targeted at their baby and not at themselves)*. A professional added: *“I think there is plenty of information everywhere but nothing is centralized, that's the problem”.*

Some professionals spoke about the pressure, when women return home, of going back to reality. Professionals seemed to understand that mothers may be lost after childbirth but thought that this mainly concerns disadvantaged women. However, it seemed to be the case for the majority of the women whatever their social background.

*“In maternity ward, there is some control, that’s reassuring. After, women are left on their own, there is a loss of control and a lack of supervision” (Focus group with professionals).*

*“They have to gain confidence in what they do. There is no need of information for that: you don’t read about it, you just need confidence” (Focus group with professionals).*

The needs of fathers after childbirth appeared to be much more organizational and practical than those of mothers.

*“We count the number of hours of sleep (Author’s note: to make sure each of them sleeps the same amount of time) and we divide daily tasks: I take care of the car and manual things; she takes care of the cooking, clothing, baby’s bag, …” (Focus group with fathers).*

For fathers, postnatal period appeared to be punctuated by the baby’s rhythm; while for mothers, it also seemed to be marked by their own pace. For fathers, as well as for mothers, the presence of the father came out as essential to support the mother. Fathers understood mothers’ need for sharing and for psychosocial support and thus were aware of the importance of continuing to have a social and family life. Paediatrician or nurse visits were also perceived as beneficial “*to ensure that all is well*”.

When we presented the needs of mothers, identified following the interviews and the focus group with mothers, to the fathers, they were quite amazed by the number and the nature of all these needs after childbirth. For fathers, the need of information seemed much less important than for mothers. They expressed the need to know what to do with the baby but added “*there is not much information, it is not always easy but we cope*”, “*I felt she had everything she needed*”. For them, fathers and mothers are in the same boat which strengthens the complicity of the couple; this did not seem to be always true for mothers. Fathers did not appear feeling the huge social pressure described by mothers but they showed to be able to understand it.

*“We understand the stress of breastfeeding: it is a lot of responsibility. If she doesn’t do it well, the baby is not well”. (Focus group with fathers).*

*“All dads should do a workshop of house dad (Author’s note: to better understand their wives)” (Focus group with fathers).*

## Discussion

The aims of this study were to identify the needs of mothers in the year following childbirth and to compare these needs between mothers who did not have the feeling of living a psychological disorder or a depression and mothers who lived a psychological disorder or had the impression of living a depression. No difference was found between mothers who experienced a psychological distress and those who did not. All women seemed to have the similar needs but at different level of intensity.

A lack of overall information, felt by mothers, after delivery, came out of this study. Indeed, the need of information during the postnatal period has already been proved in several studies [13–17]. Primiparous women felt this lack very strongly in many perspectives (medically, administratively, regarding existing services, etc.). The need for information appeared reduced and more specific for subsequent children. Indeed, mothers who already lived a maternity experience rather seek information on what they have not already experienced with their first child. Nevertheless, they would have liked more information for their first child. New mothers felt unpreparedness for the realities of motherhood (especially women having their first baby) [15, 18, 19] and search for reliable and realistic information.

Prenatal care – single, in couple or in groups – seems to help mothers to meet their need of information. Indeed, they say that, as they do not know what to expect, they need people to anticipate, for them, the questions and needs they might encounter; especially for a first baby. Women are enthusiastic about the idea of group prenatal care not only because of a favourable social climate and facilitated group discussions but also because the group leader is a valuable source of information about pregnancy and postpartum [24, 25]. Being with other people, women benefit from hearing others’ questions as well as the professional responses to those questions. However, women would like more postpartum issues to be discussed in order to be better prepared. For them, these questions should be raised and addressed in the last two months of pregnancy. Moreover, some of these group prenatal cares (e.g. yoga, relaxation therapy, aromatherapy) also demonstrated a preventive effect against maternal anxiety and depression [26–29]. It is to note that women would like this dynamic to continue in the postpartum period (e.g. group of postnatal gymnastics).

The needs of psychological support and to share experience have also already been demonstrated in the literature. Indeed, women often have a lot of fears and anxieties around the early motherhood and their changing role [4]. A study showed that, during the first year after childbirth, mothers felt lack of control over their lives, incomplete maternal feelings and unstable relation to their husbands and others [18]. Mothers seems to experience an important paradox: they are happy to be mothers but unhappy at the losses that early motherhood inflicts upon their lives (losses of autonomy and time, appearance, femininity and sexuality, and occupational identity) [30]. Women therefore need to be surrounded, reassured and understood by those who will emotionally support them in this difficult period of life [5, 6]. This study showed that mothers liked to have the possibility of discussing issues with other mothers, especially to find out if what they are experiencing is normal. The concept of normality seems very important in this period of life. Mothers denounce a huge idealization of motherhood and say that they are unable to assume it. Indeed, they say that everything (images, photos, society, other mothers ...) makes them believe that becoming a mother is only happiness. Therefore, when a mother is not well or when she had enough, she does not dare to admit it. There is such a taboo surrounding this issue that every woman feels unique in her distress, believes to be abnormal or feels guilty at the thought of not being happy or not to love her baby. There is a tremendous social pressure on women and mothers. Mothers would like to be warned of the difficulty of becoming a mother. They would need to be told that they must dare to ask for help (housework, babysitting, psychological ...); that they are not alone in this situation and certainly not the only one; and that they have to accept to be overwhelmed during this adjustment period, which can be short or long depending on the individual.

One way for mothers to deal with this fear of abnormality resides in the knowledge of what other mothers live. Sharing experiences seems to be a very important step in the appropriation of the role of mother. They can compare their experience with that of others and determine whether they do well or not. It seems that mothers want people around them to be empathetic and understand that it is difficult to become a mother. They need a permanent psychological support. The father’s place is paramount. Mothers need him to be present and to live, with them, this stage of their new life. Once again, group prenatal cares can help fill those needs of sharing experience and of psychological support [24, 25]. Nevertheless, even with those group prenatal cares, mothers feel isolated after childbirth. They would like to see friends or to have group sessions organized with other mothers (either for specific activities or simply for discussion).

The isolation problem seems quite recurring. Many women reported feeling loneliness after delivery. They spoke of the feeling that everything revolved around them during the nine months of pregnancy but that after childbirth, it stopped and everything revolved around their new baby. Some women lived this feeling as a rejection or as being abandoned, which added to the difficulty already encountered in their adaptation process. Russo et al. described the feeling of loneliness and isolation among immigrant women who were away from their families [31] but it seems that it affects a much larger population of women. Maintaining a social life is therefore paramount in the postpartum period. Sharing experience and psychological support by family and friends helps to fight against the feeling of loneliness. Home visits by a nurse or organisation of activities between mothers [24, 25] also appear like solutions.

The desire of continuity in the follow-up care also emerged from this study. Indeed, women have repeatedly expressed the desire to have a reference person who would follow them during pregnancy and after childbirth. Since they are in a difficult transition period, they believe that this system of having a reference person who they could trust and to whom they could refer would help them tremendously. This system would also have the advantage of having one and only opinion on the different issues encountered and/or to be able to discuss the various, and often diverging, advices heard, with that person. This need for continuity has already been shown in several studies [32–35].

### Strength and limitations of the study

To the best of our knowledge, this study is the first to evaluate the mothers’ needs during the postnatal period and to compare the needs of mothers with and without an experience of psychological distress. The qualitative method was well adapted to explore the mothers’ needs and to obtain very rich results. This study also explored – for the focus groups – some original co-creating methods which were drafted and exploited in collaboration with a group of experts for the co-created study design. The results were analysed by to independent researchers which allowed a strong credibility of the results. The methods were therefore rigorous.

Our study also presented some possible limitations. Firstly, the sample is composed of voluntary participants which can limit the extrapolation of the results to all mothers in Belgium. Indeed, we could suppose that mothers who came back work or mothers who lived an intense postnatal depression were less likely to participate in our study. Nevertheless, our individual interviews’ data allowed us to arrive at the topic saturation. A second possible limitation of our study may be the representativeness of the results of the focus group. Although it was quite easy to recruit some participants for the mothers’ and the professionals’ focus group, we had great difficulties in recruiting participants for the fathers’ focus group. Indeed, we had to cancel two focus groups’ dates before the fathers’ focus group happened due to a lack of participants. We decided to organize it anyway - in spite of the fact that there were only 2 fathers - knowing that we would only have a limited opinion of fathers. The results concerning the comparison of the mothers’ needs and the fathers’ perception about the mothers’ needs should therefore be interpreted with cautious.

## Conclusion

All mothers – who experienced a psychological distress and who did not – seemed to have similar needs during the postpartum period but at different level of intensity. Indeed, young mothers, apparently, feel a lack of support at different levels in the postpartum period. Women do feel neither sufficiently informed about this difficult period of life nor sufficiently supported, not only from a psychological point of view but also from a more practical point of view, for example with household chores. They are often tired and have many questions. They need to share with family, friends and other women about this experience of life and they need to be reassured and feel understood. This study helps to understand what women experience after childbirth. By providing ways to meet women’s needs, it also offers leads to prevent the risk of psychological distress in the postpartum period. It is necessary to lift the taboo on the postnatal period and stop to idealize the motherhood. Mothers should not be afraid to talk about their needs or to ask some help if they need it. Health professionals but also fathers and mothers’ family and friends should be more attentive to the mothers’ needs.

## References

[1] Cristescu T, Behrman S, Jones SV, Chouliaras L, Ebmeier KP (2015). Be vigilant for perinatal mental health problems. [Internet]. Practitioner.

[2] Dayan J (2007). Clinical approach and epidemiological aspects of mood and anxiety disorders during pregnancy and postpartum. Review and synthesis. J Gynecol Obstet Biol Reprod.

[3] Norhayati MN, Nik Hazlina NH, Asrenee AR, Wan Emilin WM (2014). Magnitude and risk factors for postpartum symptoms: a literature review. J Affect Disord.

[4] Forster DA, McLachlan HL, Rayner J, Yelland J, Gold L, Rayner S (2008). The early postnatal period: exploring women’s views, expectations and experiences of care using focus groups in Victoria, Australia. BMC Pregnancy Childbirth.

[5] McKellar LV, Pincombe JI, Henderson AM (2006). Insights from Australian parents into educational experiences in the early postnatal period. Midwifery.

[6] Hildingsson IM (2007). New parents’ experiences of postnatal care in Sweden. Women Birth.

[7] Coates R, Ayers S, de Visser R (2014). Women’s experiences of postnatal distress: a qualitative study. BMC Pregnancy Childbirth.

[8] Vossbeck-Elsebusch AN, Freisfeld C, Ehring T (2014). Predictors of posttraumatic stress symptoms following childbirth. BMC Psychiatry.

[9] Kosińska-Kaczyńska K, Horosz E, Wielgoś M, Szymusik I (2008). Affective disorders in the first week after the delivery: prevalence and risk factors. [Internet]. Ginekol Pol.

[10] Halbreich U, Karkun S (2006). Cross-cultural and social diversity of prevalence of postpartum depression and depressive symptoms. J Affect Disord.

[11] Patel M, Bailey RK, Jabeen S, Ali S, Barker NC, Osiezagha K (2012). Postpartum depression: a review. J Health Care Poor Underserved.

[12] Benahmed N, Devos C, San Miguel L, Vinck I, Vankelst L, Lauwerier E (2014). KCE reports 232B: caring for mothers and newborns after uncomplicated delivery: towards integrated postnatal care.

[13] Slomian J, Bruyère O, Reginster JY, Emonts P (2017). The internet as a source of information used by women after childbirth to meet their need for information: A web-based survey. Midwifery.

[14] Emmanuel E, Creedy D, Fraser J (2001). What mothers want: a postnatal survey. [Internet]. Aust J Midwifery.

[15] Carolan M (2007). Health literacy and the information needs and dilemmas of first-time mothers over 35 years. J Clin Nurs.

[16] Malata A, Chirwa E (2011). Childbirth information needs for first time Malawian mothers who attended antenatal clinics. [Internet]. Malawi Med J.

[17] Sword W, Watt S (2005). Learning needs of postpartum women: does socioeconomic status matter?. Birth (Berkeley, Calif).

[18] Javadifar N, Majlesi F, Nikbakht A, Nedjat S, Montazeri A (2016). Journey to motherhood in the first year after child birth. [Internet]. J Family Reprod Health.

[19] Deave T, Johnson D, Ingram J (2008). Transition to parenthood: the needs of parents in pregnancy and early parenthood. BMC Pregnancy Childbirth.

[20] Guedeney N, Fermanian J (1998). Validation study of the French version of the Edinburgh Postnatal Depression Scale (EPDS): new results about use and psychometric properties. Eur Psychiatry.

[21] Burgess-Allen J, Owen-Smith V (2010). Using mind mapping techniques for rapid qualitative data analysis in public participation processes. Health Expect.

[22] Glaser BG, Strauss AL. The discovery of grounded theory: strategies for qualitative research [Internet]. Aldine Pub. Co, 1967, [cited 2017 Mar 29].Available from: https://books.google.co.in/books/about/The_Discovery_of_Grounded_Theory.html?id=oUxEAQAAIAAJ.

[23] Neale J (2016). Iterative categorization (IC): a systematic technique for analysing qualitative data. Addiction.

[24] Novick G, Sadler LS, Kennedy HP, Cohen SS, Groce NE, Knafl KA (2011). Women’s experience of group prenatal care. Qual Health Res.

[25] McNeil DA, Vekved M, Dolan SM, Siever J, Horn S, Tough SC (2012). Getting more than they realized they needed: a qualitative study of women’s experience of group prenatal care. BMC Pregnancy Childbirth.

[26] Newham JJ, Wittkowski A, Hurley J, Aplin JD, Westwood M (2014). Effects of antenatal yoga on maternal anxiety and depression: a randomized controlled trial. Depress Anxiety.

[27] Chen S-L, Chen C-H (2015). Effects of lavender tea on fatigue, depression, and maternal-infant attachment in sleep-disturbed postnatal women. Worldviews Evid-Based Nurs.

[28] Conrad P, Adams C (2012). The effects of clinical aromatherapy for anxiety and depression in the high risk postpartum woman - a pilot study. Complement Ther Clin Pract.

[29] Akbarzadeh M, Masoudi Z, Zare N, Vaziri F. Comparison of the effects of doula supportive care and acupressure at the BL32 point on the mother’s anxiety level and delivery outcome. [Internet]. Iran J Nurs Midwifery Res. 2015;20:239–46. [cited 2016 Feb 24] PMC4387650 25878703

[30] Nicolson P (1999). Paula: Loss, happiness and postpartum depression: the ultimate paradox. CanPsychol.

[31] Russo A, Lewis B, Joyce A, Crockett B, Luchters S (2015). A qualitative exploration of the emotional wellbeing and support needs of new mothers from Afghanistan living in Melbourne, Australia. BMC Pregnancy Childbirth.

[32] Forster DA, McLachlan HL, Davey M-A, Biro MA, Farrell T, Gold L (2016). Continuity of care by a primary midwife (caseload midwifery) increases women’s satisfaction with antenatal, intrapartum and postpartum care: results from the COSMOS randomised controlled trial. BMC Pregnancy Childbirth.

[33] Rayner J-A, McLachlan HL, Peters L, Forster DA (2013). Care providers’ views and experiences of postnatal care in private hospitals in Victoria, Australia. Midwifery.

[34] Brown SJ, Davey M-A, Bruinsma FJ (2005). Women’s views and experiences of postnatal hospital care in the victorian survey of recent mothers 2000. Midwifery.

[35] Jenkins MG, Ford JB, Todd AL, Forsyth R, Morris JM, Roberts CL (2015). Women’s views about maternity care: how do women conceptualise the process of continuity?. Midwifery.

